# Biobleaching of Industrial Important Dyes with Peroxidase Partially Purified from Garlic

**DOI:** 10.1155/2014/183163

**Published:** 2014-10-23

**Authors:** Akudo Chigozirim Osuji, Sabinus Oscar O. Eze, Emmanuel Emeka Osayi, Ferdinand Chiemeka Chilaka

**Affiliations:** ^1^Department of Biochemistry, University of Nigeria, Nsukka, Nigeria; ^2^Department of Plant Sciences and Biotechnology, University of Nigeria, Nsukka, Nigeria

## Abstract

An acidic peroxidase was extracted from garlic (*Allium sativum*) and was partially purified threefold by ammonium sulphate precipitation, dialysis, and gel filtration chromatography using sephadex G-200. The specific activity of the enzyme increased from 4.89 U/mg after ammonium sulphate precipitation to 25.26 U/mg after gel filtration chromatography. The optimum temperature and pH of the enzyme were 50°C and 5.0, respectively. The Km and *V*_max_ for H_2_O_2_ and o-dianisidine were 0.026 mM and 0.8 U/min, and 25 mM and 0.75 U/min, respectively. Peroxidase from garlic was effective in decolourizing Vat Yellow 2, Vat Orange 11, and Vat Black 27 better than Vat Green 9 dye. For all the parameters monitored, the decolourization was more effective at a pH range, temperature, H_2_O_2_ concentration, and enzyme concentration of 4.5–5.0, 50°C, 0.6 mM, and 0.20 U/mL, respectively. The observed properties of the enzyme together with its low cost of extraction (from local sources) show the potential of this enzyme for practical application in industrial wastewater treatment especially with hydrogen peroxide. These Vat dyes also exhibited potentials of acting as peroxidase inhibitors at alkaline pH range.

## 1. Introduction

The world's ever increasing population and the progressive adoption of an industrial based life style have inevitably led to an increased anthropogenic impact on the biosphere [1]. The textile and leather industries are of interest in this regard [2]. Synthetic dyes are integral part of tannery industries. Subashini et al. [3] observed that one of the major environmental problems is the presence of dye materials in textile wastewater, which need to be removed before being released into the environment. Wastewater from tanneries usually contains high concentration of dyes, neutral salts, aliphatic and aromatic polymeric substances, and polyphenols [4]. Since these dye molecules are often toxic and hard to degrade in the conventional wastewater treatment systems [5], proper treatment of the coloured effluent is of great importance. Among the textile dyes, azo dyes account for 60–70% of all textile dye stuff used and show the largest spectrum of colours [6].

A number of physicochemical treatments have been employed to treat dye-house wastewaters. These include conventional methods such as coagulation, flocculation, sorption, electrochemical decomposition, or oxidative degradation [7]. However, conventional methods are usually limited by large sludge generation, high costs, demand for an external reagent, and being tedious [8]. Hence, alternative treatment processes based on biotechnological principles have gained popularity in recent years [9]. Biocatalysis based effluent treatment has outclassed the presently favoured physicochemical treatments due to nil sludge production and monetary savings [5].

Over the last 20 years, research has concentrated on using microorganisms for the removal of colour from textile industrial wastewater [10]. Both aerobic and anaerobic microorganisms have been employed in biological colour removal [3]. The drawback in using microorganisms to degrade target recalcitrant pollutants in actual effluents is that the microbes need to get acclimatized to the effluent [4] and this process can be time consuming. Also, an additional substrate may be needed to sustain the microbial culture if the effluent does not contain metabolic substrates of the selected microorganism [11].

Recently, enzymatic approach has attracted much interest in the removal of dye from aqueous solutions [12], because enzymes can act on recalcitrant pollutants to remove them by precipitation or transformation to other products. From a practical point of view the use of peroxidases* in vitro* for this purpose may represent a more feasible system.

Peroxidases are widely distributed in plants, microorganisms, and animals where they play important roles. Plant peroxidases (EC 1.1.11.7) are hemoproteins that catalyse the H_2_O_2_ dependent oxidation of a wide variety of substrates including phenolic compounds [13]. Reduction of peroxides at the expense of electron donating substrates makes peroxidases useful in a number of biotechnological applications such as biopulping and biobleaching in the paper industry and can produce oxidative breakdown of synthetic azo dyes [14]. Peroxidases from plant sources, horseradish, turnip, tomato, soybean, bitter gourd, white radish, and* Saccharum uvarum,* have been employed for the remediation of commercial dyes [15]. Garlic,* Allium sativum*, is known to contain thermostable peroxidase and has been employed in the detection of H_2_O_2_ in milk. Keeping in mind the dearth of information on* Allium sativum* peroxidase, its use in industrial wastewater treatment, and the significance of plant peroxidases, the present study has been undertaken to investigate the decolourization potential of the* Allium sativum* peroxidase for Vat dyes.

## 2. Materials and Methods

Peeled garlic (100 g) was homogenized in 200 mL of 0.05 M phosphate buffer pH 6.5 using the Omni general laboratory homogenizer (GLH) and kept at 29°C for 24 h with constant stirring on MRAD Corporation mechanical shaker 311 series at low speed. The homogenate was filtered with double-layered cheesecloth. The filtrate was centrifuged with Cole-Palmer VS-13000 microcentrifuge at 4000 rmp for 30 min. The supernatant was collected and stored at 10°C as crude enzyme extract.

Protein content of the crude enzyme extract was determined by the method of Lowry et al. [16] using Bovine serum albumin as standard unless otherwise stated.

Peroxidase activity was assayed using the method of Eze et al. [17] with slight modification. The assay mixture contained 2.4 mL of 0.05 M sodium phosphate buffer pH 6.0, 0.2 mL of 0.8% H_2_O_2_, 0.2 mL of 1% o-dianisidine, and 0.2 mL of the crude enzyme. Peroxidase activity was monitored by change in absorbance due to oxidation of o-dianisidine in the presence of hydrogen peroxide using Jenway 6405 UV/VIS spectrophotometer.

The crude enzyme extract was partially purified by ammonium sulphate saturation up to 80%, stirred for about 6 h using an STI Cole-Palmer magnetic stirrer, and kept at 2°C for 24 h. This was centrifuged at 10,000 rmp with Thermo Scientific Heraeus Primo/Primo R centrifuge for 30 min. The precipitate was dissolved in 0.05 M sodium phosphate buffer pH 6.0 and dialyzed for 18 h against the same buffer.

The dialysate was applied to a sephadex G-200 column (2.5 cm × 50) preequilibrated with 0.01 M phosphate buffer, pH 6.0, and then eluted with about 500 mL of 0.01 M sodium phosphate buffer. The fractions that showed high peroxidase activity were pooled together. Protein concentration of the eluents was monitored by following the absorbance at 280 nm using Jenway 6405 UV/VIS spectrophotometer.

The optimum pH for peroxidase activity was determined by monitoring the activity of the enzyme as in the assay section using the following buffers: 0.05 M sodium acetate buffer (pH 3.5–5.5), 0.05 M phosphate buffer (pH 6.0–7.5), and 0.05 M Tris-HCl buffer (pH 8.0–9.5).

The optimum temperature was determined by assaying for the activity of the enzyme as in the assay section at different temperatures (30–70°C).

The Km and *V*_max⁡_ for hydrogen peroxide and o-dianisidine for* Allium sativum* peroxidase were determined as follows: different concentrations of H_2_O_2_ (0.05–1 mM) (in triplicate) were used to make assay for the activity of peroxidase as described in the assay section. The average of the data generated from the assay was used to construct the Lineweaver-Burk plot from which the Km and *V*_max⁡_ were determined for H_2_O_2_. Also different concentrations of o-dianisidine (20–120 mM) were prepared (in triplicate) and used to make assay for peroxidase activity as contained in the assay section and treated as in that of hydrogen peroxide above.

Dye decolourization with garlic peroxidase was carried out using the method described by Jamal et al. [18]. In this experiment, the effect of varying the pH, temperature, enzyme, and hydrogen peroxide concentration, respectively, on the decolourization of the dyes by the* Allium sativum* peroxidase was determined. The following Vat dyes were used for this study (Vat Yellow 2, Vat Orange 11, Vat Green 9, and Vat Black 27). The activity of garlic peroxidase on each of the vat dyes was determined in a reaction mixture which contains 2.2 mL of 0.05 M phosphate buffer pH 6.0, 0.4 mL of the 0.1% dye solution (different dyes one at a time), 0.2 mL of H_2_O_2_, and 0.2 mL of the enzyme in a total of 3 mL. Each of the dyes was incubated differently with the reaction mixture for a period of 4 h at 50°C in an MRC stainless steel water bath, model WBO-200 and then centrifuged at 4000 rpm using Thermo Scientific Sorvall ST 8 bench top centrifuge for 10 min and absorbance was read (before and after incubation) at 460, 480, 600, and 680 nm for Vat Yellow 2, Vat Orange 11, Vat Green 9, and Vat Black 27, respectively. The percentage dye decolourization was calculated thus as follows:

(1) $$\begin{array}{c} \%\;\text{decolourization}=\frac{A_{i}-A_{f}}{A_{i}}\times \frac{100}{1}, \end{array}$$

where *A*_*i*_ is the absorbance before incubation and *A*_*f*_ is the absorbance after incubation.

Also the effect of different concentration of dye on its decolourization by the enzyme was studied. The reaction mixture consists of the cocktail as in the assay section but with different concentration of the dye (0.1–2 *μ*M) in each of the reaction mixtures in a total of 3 mL. The percentage decolourization was calculated.

Effects of temperature on dye decolourization were monitored by incubating the reaction mixtures (as in assay section) in an MRC stainless steel water bath model WBO-200 at varying temperatures (30–75°C) after which the percentage dye decolourized was calculated as earlier described. For temperature stability, enzyme solutions were incubated at 30–80°C for 3 h and then cooled at 0°C for another 30 min; the enzyme residual activity was determined as earlier described.

The effect of pH on the decolourization of Vat dyes by garlic peroxidase was monitored by incubating the reaction mixture (as in assay section) using buffers of different pHs; 0.05 M acetate buffer (pH 3.5–5.5); 0.05 M sodium phosphate buffer (pH 6.0–7.5); and 0.05 M Tris-HCL buffer (pH 8.0–10) at 50°C for 4 h. At the end of incubation, the percentage decolourization was calculated as earlier described. pH stability was determined by incubating the enzyme solution in various buffers of different pHs between 3.0 and 10.0 (as mentioned above) for 1 h at room temperature and thereafter, the enzyme residual activity was monitored as earlier described.

Different concentrations of H_2_O_2_ (0.2 to 1.6 mM) were also used to make assay for the decolourization of Vat dyes using garlic peroxidase.

Varying concentrations of garlic peroxidase (0.04–0.32 U/mL) were incorporated in the reaction mixtures (as in assay section) and incubated as earlier described. The percentage dye decolourization was calculated.

Data were analyzed by analysis of variance (ANOVA) using the statistical package for social solutions (SPSS) version 2.0. Difference of means was considered significant at *P* < 0.05.

## 3. Results and Discussion

**Figure 1:**
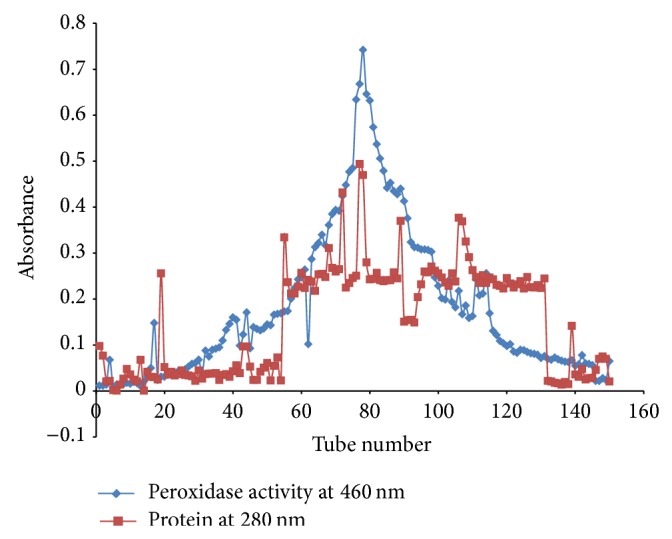
Gel filtration chromatography elution profile.

Figure 1 shows the elution profile of garlic peroxidase on sephadex G-200 gel filtration chromatographic column. The fractions containing high peroxidase activity whose peak coincided with protein peak (tube 79) were used for enzyme characterization and decolourization studies.

The protein concentration of the crude extract was found to be 3.981 mg/mL which rose to 5.669 mg/mL after ammonium sulphate precipitation indicating that much of the protein was precipitated. After 18 h of dialysis, the protein concentration was reduced to 2.650 mg/mL and further to 2.068 mg/mL after gel filtration. An enzyme activity of 20.39 U, 27.70 U, 41.78 U, and 52.23 U was observed for the crude enzyme, ammonium sulphate precipitated enzyme, dialyzed enzyme, and the enzyme resulting from gel filtration, respectively. Peroxidase activity increased simultaneously with increase in purification steps (Table 1). The specific activity of the crude enzyme was found to be 4.094 U/mg. This value increased to 4.886 U/mg, 15.766 U/mg, and 25.256 U/mg after ammonium sulphate precipitation, dialysis, and gel filtration, respectively. The increase in specific activity is a measure of purification achieved. Peroxidase extracted from garlic* (Allium sativum*) was purified approximately threefold.

**Figure 2:**
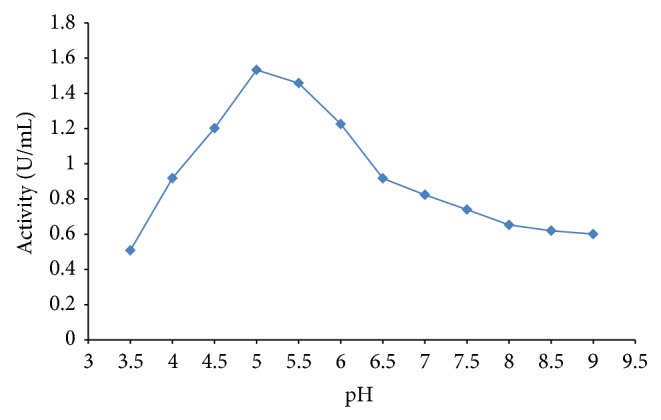
Effect of pH on peroxidase activity.

**Figure 3:**
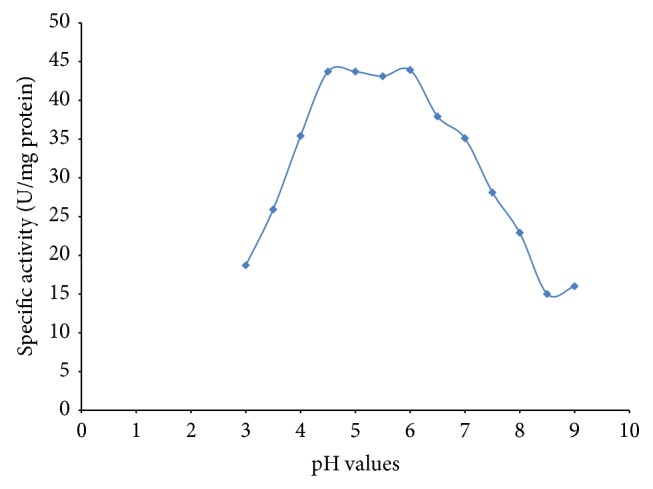
pH stability of garlic peroxidase.

From the pH studies, as the pH was increased, from pH 3.5 to pH 5.0, the peroxidase activity was found to increase. Further increase in pH resulted in a decrease of peroxidase activity (Figure 2). Peroxidase activity was found to be pH-dependent with inactivation observed within the alkaline pHs. Mizobutsi et al. [19] reported pH optimum of 6.5 for peroxidase from* Litchi* fruit. Mohapatra [4] reported an optimum pH of 4.0 for peroxidases from* S. bicolor* using guiacol as the substrate. Haq et al. [20] also reported an optimum peroxidase activity at pH 4.0 from* Raphanus sativus*. The result of this study is consistent with the findings of Marzouki et al. [21] who reported an optimum pH of 5.0 for peroxidase from* Allium sativum*. It is also comparable with the pH optimum of 5.0 reported by Kim and Lee [22] for peroxidase from sunflower. Garlic peroxidase was also found to be stable between pH 4.0 and 6.0 (Figure 3).

**Figure 4:**
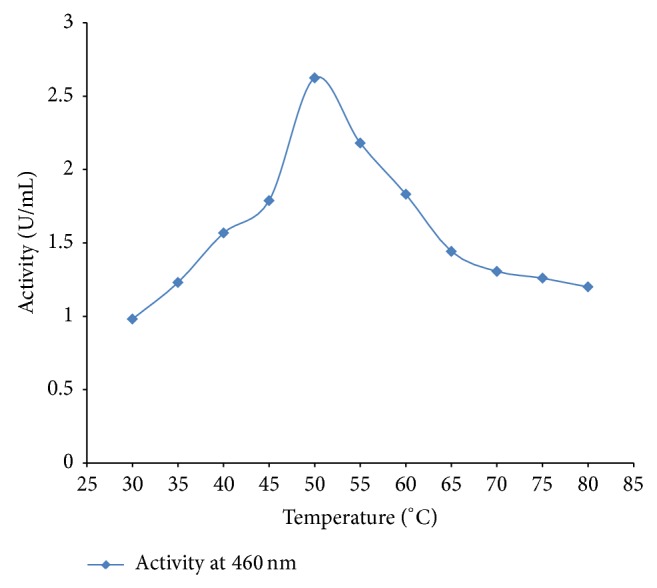
Effect of temperature on peroxidase activity.

**Figure 5:**
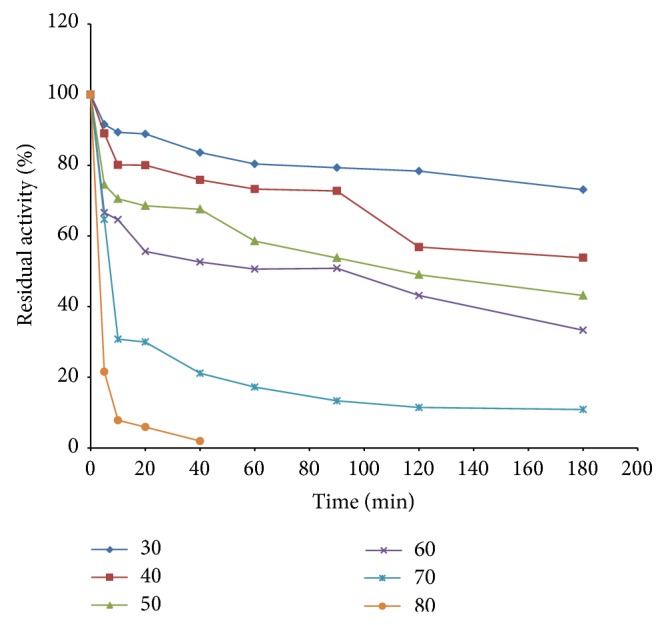
Heat stability of garlic peroxidase.

Figure 4 shows an optimum temperature of 50°C for the enzyme. When the temperature exceeds 50°C, the activity began to decline. Haq et al. [20] recorded an optimum activity at 55°C for peroxidase from* Raphanus sativus*. Onder et al. [23] reported an optimum temperature of 30°C for peroxidase from Turkish black radish using guiacol as substrate. Mamounata et al. [24] recorded an optimum temperature range of 30–40°C for peroxidases from* Raphanus sativus, Allium sativum, Ipomoea batatas, and Sorghum bicolor* grown in Burkina Faso. Matto and Husain [25] observed an optimum temperature of 50°C for cabbage peroxidase and 40°C for radish and tobacco peroxidases. The result of this study is also comparable with the optimum temperature reported by Omidiji et al.[26] for peroxidase from* Dioscorea esculenta L.* tubers. The enzyme was found to be less thermostable at higher temperatures (Figure 5). It was found to be thermostable at 40, 50, and 60°C, respectively. The thermostability dropped drastically at 70 and 80°C within 10 min of heat treatment (Figure 5) suggesting that high temperature short time treatment could easily inactivate the enzyme.

**Figure 6:**
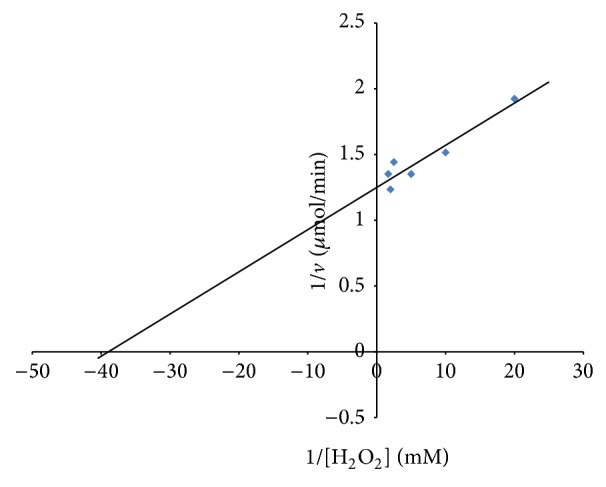
Lineweaver-Burk plot of initial velocity data using different concentration of H_2_O_2_ as substrate.

**Figure 7:**
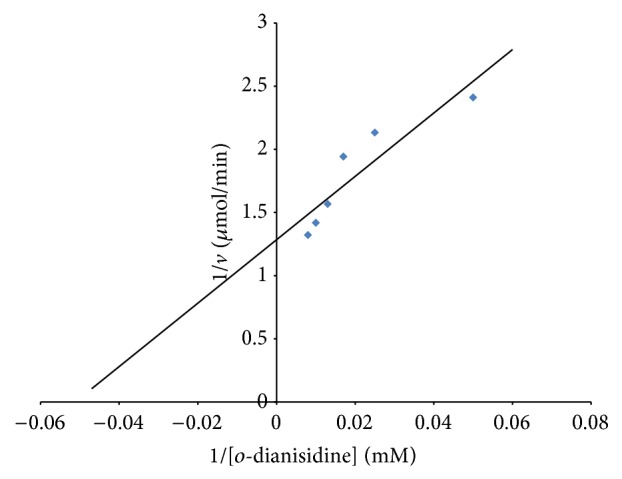
Lineweaver-Burk plot of initial velocity data using different concentration of o-dianisidine as substrate.

Figures 6 and 7 show the Lineweaver-Burk plots for the initial velocity data using hydrogen peroxide and o*-*dianisidine, respectively, as substrates. The Km and *V*_max⁡_ for hydrogen peroxide as substrate were observed to be 0.026 mM and 0.8 U/min, respectively, while the Km and *V*_max⁡_ of 25 mM and 0.79 U/min were obtained using o*-*dianisidine as substrate. Onder et al. [23] reported a Km of 1.18 mM and *V*_max⁡_ of 0.032 U/min for peroxidase from* Raphanus sativus *L. when hydrogen peroxide was used as substrate.

**Figure 8:**
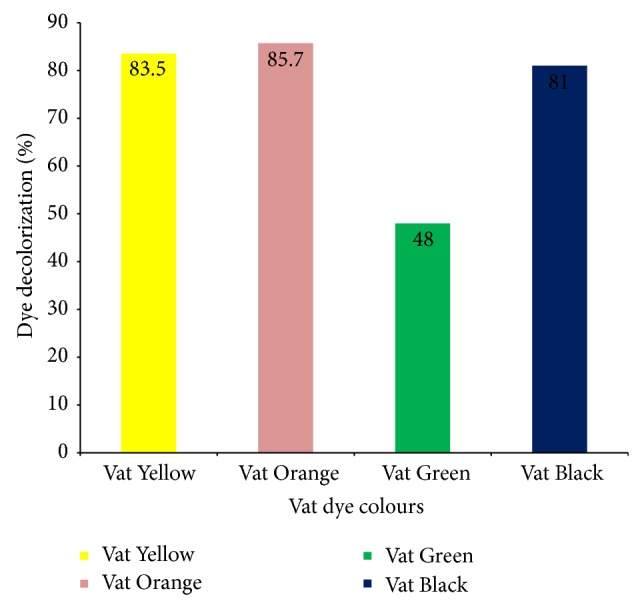
Percentage dye decolourization after 4 h of incubation.

The decolourization potential of garlic peroxidase was tested using four Vat dyes—Vat Yellow 2, Vat Orange 11, Vat Green 9, and Vat Black 27. After 4 h of incubation in an MRC stainless steel water bath model WBO-200 at 50°C (Figure 8), the percentage decolourization for each dye was found to be 83.5, 85.6, 48, and 81% for Vat Yellow 2, Vat Orange 11, Vat Green 9, and Vat Black 27, respectively. Subramaniam et al. [27] reported 23% decolourization of tannery effluent dyes using* Momordica charantia* peroxidase, after 4 h of incubation at 50°C. Onder et al. [23] recorded 90% decolourization of naphthol blue after 5 min of contact time at the temperature of 40°C using horseradish peroxidase. Husain et al. [28] recorded 85% decolourization of textile effluent dyes after 5 h incubation at 40°C using* fenugreek* peroxidase. The differences in time course of removal of these dyes as reported by various scholars might be due to the structural barrier and electron localization among the dyes. Also, the level of purification of the peroxidase used for biobleaching is an important factor to be considered.

**Figure 9:**
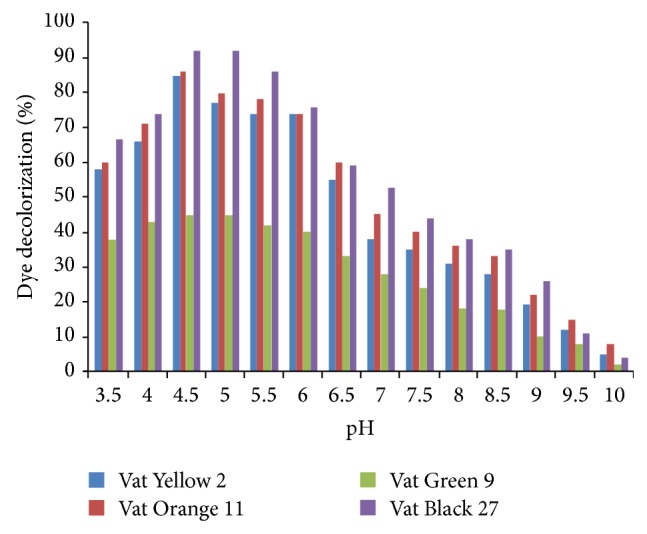
Dye decolourization as a function of pH.

Effect of pH on the decolourization of the dyes was monitored at a pH range of 3.5–10.0. The highest percentage decolourization was observed at pH 4.5 for all the dyes used at 85, 86, 45, and 92% for Vat Yellow 2, Vat Orange 11, Vat Green 9, and Vat Black 27, respectively (Figure 9). At pH 5 and pH 5.5, the decrease in percentage decolourization percentage was not very significant (*P* > 0.05). A significant decrease (*P* < 0.05) was noticed from pH of 6.5 down to the alkaline pH as shown in Figure 9. Vasantha et al. [29] reported the best decolourization of Direct Yellow 12 using horseradish peroxidase at pH of 4.0. Husain et al. [28] observed 68% decolourization of industrial effluent dyes at pH 5.0 using peroxidase from fenugreek. Literature has shown that the highest/best percentage decolourization of various synthetic dyes occurs within pH 4–6.0. The result of this investigation which shows a pH of 4.5 as the best pH for the decolourization of the various dyes under study is in agreement with literature.

From our result, one can infer that Vat dyes under investigation acted as inhibitors of garlic peroxidase at alkaline pH. This could open up research on the possible use of these Vat dyes as possible peroxidase inhibitors.

**Figure 10:**
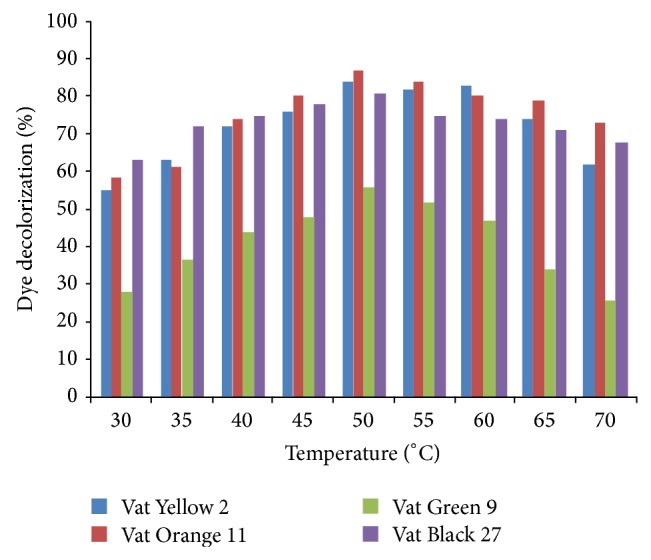
Effect of temperature on dye decolourization with garlic peroxidase.

Figure 10 shows that, after 4 h of incubation of the Vat dyes with garlic peroxidase at different temperatures (30–70°C), the percentage of dyes decolourized at 50°C is 84, 87, 56 and 81% for Vat Yellow 2, Vat Orange 11, Vat Green 9 and Vat Black 27 respectively making it the best temp for these dyes to be decolourized. Haq et al. [20] also observed the best percentage decolourization of 97 and 77 for Solar Blue A and Solar Flavine 5G dyes at 50°C using* Raphanus sativus* peroxidase.

Above and below the optimum temperature 50°C, the rate of decolourization decreased. The decrease in percentage decolourization at higher temperatures might be due to the denaturation of the enzyme at higher temperatures which results in low enzyme activity and hence low decolourization rate.

**Figure 11:**
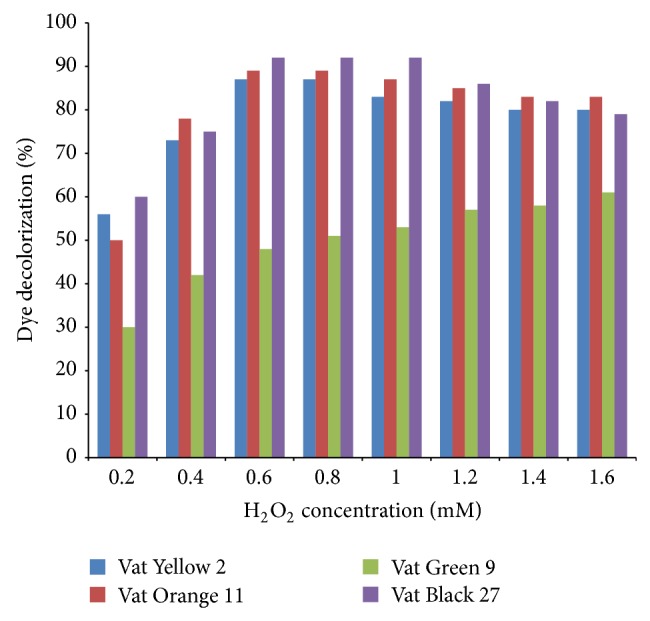
Effect of different concentrations of H_2_O_2_ on dye decolourization with garlic peroxidase.

Figure 11 shows the effect of different concentration of H_2_O_2_ on dye decolourization. Different concentrations of hydrogen peroxide from 0.2 to 1.2 mM were used. The dye was best decolourized at a concentration of 6 mM H_2_O_2_ with 87, 89, and 92% decolourization for Vat Yellow 2, Vat Orange 11, and Vat Black 27 dyes, respectively. On the contrary, the Vat Green 9 had its best decolourization of 48% at 2.2 mM concentration of hydrogen peroxide. This could be because of the complex structure of the Vat Green 9 dye which is synthesized based on the structure of violanthrones known for their complex structure, while Vat Yellow 2, Vat Orange 11, and Vat Black 27 are synthesized based on the anthraquinone carbazoles, known for their simple structure as reported by Aspland [30]. Husain [15] reported that at the concentration of hydrogen peroxide greater than 0.75 mM, peroxidase activity was inhibited by irreversibly oxidizing the enzyme ferriheme group, essential for peroxidase activity. In this study we observed a relative lower concentration of hydrogen peroxide. The result of this study is in agreement with that of Vasantha et al.[29] who reported a concentration of 0.6 mM H_2_O_2_ for various dyes treated. This result is also in agreement with the report of Vazquez-Duarte et al. [31] who reported that the concentration of hydrogen peroxide greater than 1.2 mM acted as inhibitor of peroxidase activity possibly by causing irreversible oxidation of the enzyme ferriheme group which is essential for its activity.

**Figure 12:**
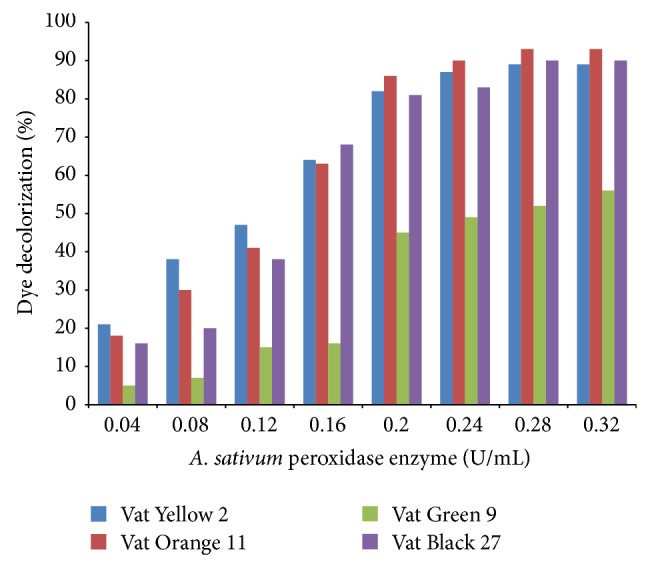
Effects of enzyme concentration on dye decolourization.

Figure 12 shows the effect of enzyme concentration on the percentage of dyes decolourized. Different concentrations of the enzyme (0.04 *μ*g/mL–0.32 *μ*g/mL) were used for the study as in the experimental section. Normally, removal of dye is dependent on the amount of catalyst added. Peroxidase activity was increasing with increase in the concentration of the enzymes till it is saturated at 0.20 *μ*g/mL for Vat Yellow 2, Vat Orange 11, and Vat Black 27, after which further increase in the concentration shows no other corresponding increase in the rate of decolourization of dye. For the Vat Green 9, decolourization of dye increased with increase in enzyme concentration till 0.23 *μ*g/mL.

**Figure 13:**
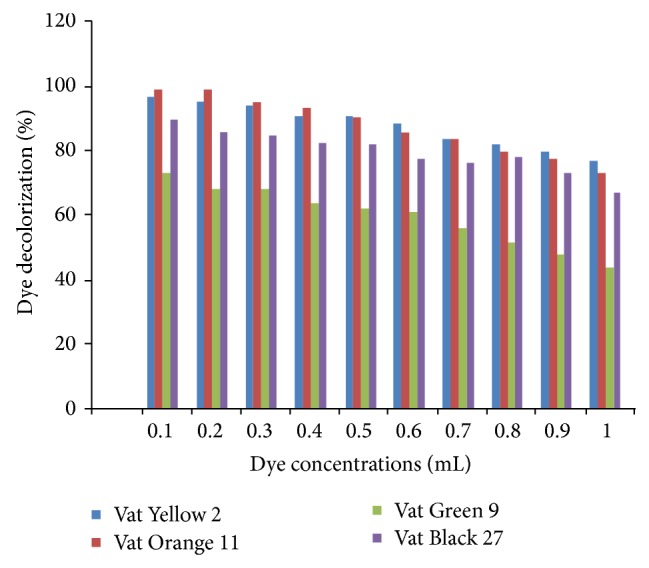
Percent decolourization as a function of dye concentration.

The concentration of the substrate present in the aqueous phase significantly influences enzyme-mediated reactions. The effect of decolourization of the 1% dye prepared as a function of volume of the dye (0.1–1 mL) was monitored by using different volumes of the dye. It was observed that percentage of dye decolourized decreased with increase in dye concentration (volumes) (Figure 13).

## 4. Conclusion

Garlic peroxidase efficiently decolourized different types of Vat dyes. For all the parameters monitored, the decolourization was more effective at a pH range, temperature, H_2_O_2_ concentration, and enzyme concentration of 4.5–5.0, 50°C, 0.6 mM, and 0.20 U/mL, respectively. This suggests that garlic peroxidase has the potential of application in environmental biotechnology and also exposes the inhibitory activities of Vat dyes on garlic peroxidase under alkaline pHs.

## Figures and Tables

**Table 1:** Purification table of garlic peroxidase.

Purification step	Volume of enzyme (mL)	Protein conc. (mg/mL)	Peroxidase activity (U/mL)	Specific activity (U/mg)	Total activity (U)	Purification fold	% yield
Crude enzyme	2000	4.981	20.39	4.09	40,780	1	100
(NH_4_)_2_SO_4_ precipitation	300	5.669	27.70	4.89	8,310	1.2	20.4
Dialyzed enzyme	180	2.650	41.78	15.77	7,520	3.9	90.5
Gel filtration	30	2.068	52.23	25.26	1,567	6.2	21
